# Une polyradiculonévrite inflammatoire démyélinisante chronique paranéoplasique secondaire à un lymphome *natural killer* nasal

**DOI:** 10.11604/pamj.2020.36.303.17772

**Published:** 2020-08-18

**Authors:** Selim Jennane, Nawal Hasnaoui, Mahtat El Mehdi, Hicham El Maaroufi, Nezha Messaoudi, Mohamed Mikdame, Kamal Doghmi

**Affiliations:** 1 Service d´Hématologie Clinique, Hôpital Militaire d´Instruction Mohammed V, Université Mohammed V, Rabat, Maroc,; 2 Laboratoire d´Hématologie, Hôpital Militaire d´Instruction Mohammed V, Université Mohammed V, Rabat, Maroc

**Keywords:** Lymphome, polyradiculonévrite, paranéoplasique, Lymphoma, polyradiculoneuritis, paraneoplastic

## Abstract

Nous rapportons un cas unique d´une polyradiculonévrite inflammatoire démyélinisante chronique paranéoplasique secondaire à un lymphome non hodgkinien T de type natural killer nasal.

## Abstract (en)

We here report a single case of chronic paraneoplastic inflammatory demyelinating polyradiculoneuritis secondary to a nasal natural killer (NK) non-Hodgkin T lymphoma.

## Introduction

La polyradiculonévrite inflammatoire démyélinisante chronique (PIDC) paranéoplasique est une complication rare décrite dans différentes pathologies néoplasiques. Son association avec un lymphome non hodgkinien T (LNHT) de type *natural killer* (NK) nasal n´a jamais été rapporté par la littérature.

## Patient et observation

Un patient de 52 ans, sans antécédent, a été admis dans notre formation pour l´exploration d´une altération de l´état général. A l´interrogatoire le patient présentait depuis trois mois, une faiblesse symétrique des muscles proximaux et distaux des quatre membres, d´aggravation progressive, ainsi que des douleurs neuropathiques au niveau des membres inférieurs. Il se plaignait aussi de l´apparition récente d´une dysphagie et d´une dysphonie ainsi qu´une perte de poids estimée à 10 kg en 3 mois. L´examen neurologique retrouvait un déficit moteur symétrique des muscles proximaux et distaux des quatre membres. La sensibilité était altérée et les réflexes ostéo-tendineux étaient absents aux membres inférieurs et diminués au niveau des membres supérieurs.

**Figure 1:**
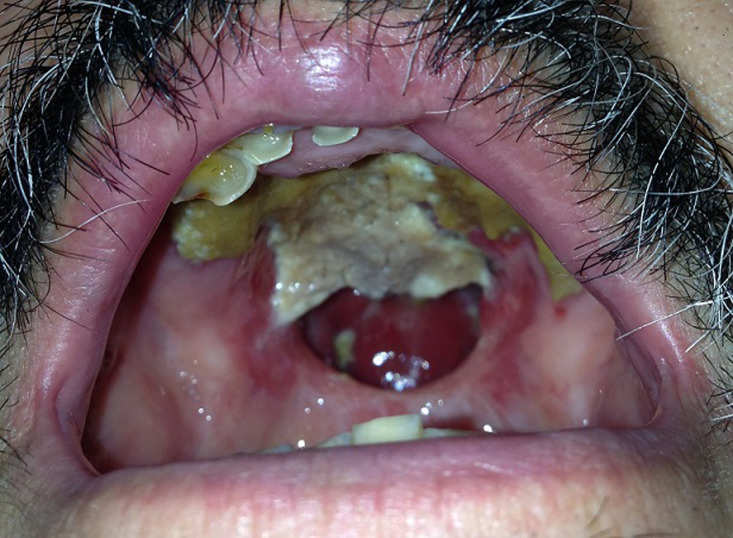
masse nécrosée, détruisant le palais osseux et créant une communication entre la cavité nasale et la cavité buccale

L´examen des nerfs crâniens était sans anomalie. L´examen de la cavité buccale montrait la présence d´une masse nécrosée qui perforait le palais dure et qui entrainait une communication entre la cavité nasale et la cavité buccale (Figure 1). L´électro neuro myogramme réalisé sur plusieurs nerfs décelait l´existence d´un bloc de conduction partiel des nerfs moteurs, un ralentissement de la vitesse de conduction des nerfs moteurs ainsi qu´un allongement de la latence motrice distale et des latences des ondes F. La ponction lombaire montrait une hyper protéinorachie à 3g/l sans hyper leucorachie (4 cellules/mm^3^). La biopsie de la masse nasale était en faveur d´un lymphome non hodgkinien T de type NK nasal. Le bilan d´extension ne retrouvait pas de localisation secondaire. L´imagerie par résonnance magnétique (IRM) cérébrale était sans anomalie. Les sérologies de l´hépatite B et C et du virus d´immunodéficience humaine (VIH) étaient négatives. La fonction rénale et le bilan hépatique étaient normaux.

Il n´existait pas de gammapathie monoclonale à l´électrophorèse des protéines sériques. Le diagnostic retenu était celui d´une polyradiculonévrite inflammatoire démyélinisante chronique paranéoplasique secondaire à un LNHT de type NK nasal. Le patient a reçu deux cures de chimiothérapie comportant la dexaméthasone, la L-asparginase ainsi que le méthotrexate entrainant une amélioration rapide mais partielle du déficit neurologique. Il a ensuite bénéficié d´une radiothérapie locale suivie de deux autres cures de chimiothérapie selon le même protocole. L´évolution était marquée initialement par l´obtention d´une réponse partielle estimée à 90% selon les critères de Cheson *et al*. [1]. A 3 mois de la fin du traitement le patient a présenté une progression du volume de la masse ainsi qu´une aggravation de la symptomatologie neurologique. Il est décédé à 11 mois du diagnostic.

## Discussion

Selon notre recherche sur « PubMed », notre patient est le premier cas décrit dans la littérature d´une PIDC secondaire à un LNHT de type NK nasal. Les PIDC sont un groupe hétérogène de neuropathies démyélinisantes auto-immunes acquises [2]. L´origine auto immune des PIDC a été évoquée par la présence des lymphocytes T et d´un infiltrat macrophagique phagocytant les débris myéliniques, dans l´endonèvre des biopsies nerveuses [3]. Ce phénomène a été ensuite conforté à l´aire des anticorps par la mise en évidence d´anticorps dirigés contre les différentes structures nerveuses [4]. Comme dans notre observation le diagnostic repose sur un faisceau d´arguments cliniques, biologiques et électrophysiologiques. Les critères utilisés actuellement sont ceux du groupe de travail conjoint de l´European Federation of Neurological Societies (EFNS) et de la Peripheral Nerve Society (PNS) [5].

Les PIDC sont souvent idiopathiques [6]. Dans certains cas, elles peuvent être secondaires ou accompagner diverses pathologies. Les causes infectieuses sont dominées les hépatites C et B, le VIH et la maladie de Lyme [6]. Parmi les maladies systémiques associées à la PIDC on distingue le lupus, la sarcoïdose, les thyroïdites auto immunes, les hépatites auto-immunes, la cryoglobulinémie, la polyarthrite rhumatoïde et le syndrome de Gougerot Sjögren [6]. En présence d´une gammapathie monoclonale, il est indispensable de vérifier si celle-ci possède une activité anticorps (anti-MAG, anti-GQ1b) et de rechercher une maladie de Waldenstrom, un myélome multiple, un syndrome de POEMS (Polyneuropathy, Organomegaly, Endocrinopathy, Monoclonal protein and Skin changes), avant de conclure à une gammapathie monoclonale de signification indéterminée (MGUS) [7, 8]. Le lien entre le PIDC et certaines pathologies reste controversé comme chez les patients atteints d´un diabète [9], d´une maladie inflammatoire chronique de l'intestin (MICI) et de certaines vascularites [7]. Une cause néoplasique doit être évoquée devant un amaigrissement, des signes généraux ou un syndrome tumoral. Plusieurs cancers solides ont été incriminés dans les PIDC dont les cancers pulmonaires, les mélanomes et les lymphomes hodgkinien et non hodgkinien [10, 11]. En plus de l´atteinte du système nerveux central par l´envahissement tumorale, les lymphomes B et T peuvent être responsable de diverses neuropathies périphériques par des mécanismes immunologiques mal élucidés [12, 13]. Les lymphomes B sont plus souvent responsables de neuropathie périphériques que les LNHT [10].

Sur le plan thérapeutique, notre choix était basé sur les études récentes montrant la supériorité des protocoles de chimiothérapie comportant la L-asparginase couplés à une radiothérapie en « sandwich » [14]. L´état général du patient ne permettait pas l´utilisation d´un protocole plus agressif.

## Conclusion

Au cours d´une hémopathie maligne, le diagnostic d´une PIDC paranéoplasique doit être évoqué devant tout déficit moteur ou sensitif et confirmé par un faisceau d´arguments cliniques, biologiques et électromyographiques.

## References

[1] Cheson BD, Pfistner B, Juweid ME, Gascoyne RD, Specht L, Horning SJ (2007). Revised response criteria for malignant lymphoma. J Clin Oncol.

[2] Rajabally YA, Simpson BS, Beri S, Bankart J, Gosalakkal JA (2009). Epidemiologic variability of chronic inflammatory demyelinating polyneuropathy with different diagnostic criteria: study of a UK population. Muscle Nerve.

[3] Bouchard C, Lacroix C, Planté V, Adams D, Chedru F, Guglielmi JM (1999). Clinicopathologic findings and prognosis of chronic inflammatory demyelinating polyneuropathy. Neurology.

[4] Mathey EK, Park SB, Hughes RA, Pollard JD, Armati PJ, Barnett MH (2015). Chronic inflammatory demyelinating polyradiculoneuropathy: from pathology to phenotype. J Neurol Neurosurg Psychiatry.

[5] Joint Task Force of the EFNS and the PNS (2010). European Federation of Neurological Societies/Peripheral Nerve Society Guideline on management of chronic inflammatory demyelinating polyradiculoneuropathy: report of a joint task force of the European Federation of Neurological Societies and the Peripheral Nerve Society? first revision. J Peripher Nerv Syst.

[6] Viala K (2007). Epidemiological and clinical aspects of CIDP. Rev Neurol (Paris).

[7] French CIDP Study group (2008). Recommendations on diagnostic strategies for chronic inflammatory demyelinating polyradiculoneuropathy. J Neurol Neurosurg Psychiatry.

[8] Viala K, Maisonobe T, Stojkovic T, Koutlidis R, Ayrignac X, Musset L (2010). A current view of the diagnosis, clinical variants, response to treatment and prognosis of chronic inflammatory demyelinating polyradiculoneuropathy. J Peripher Nerv Syst.

[9] Kalita J, Misra UK, Yadav RK (2007). A comparative study of chronic inflammatory demyelinating polyradiculoneuropathy with and without diabetes mellitus. Eur J Neurol.

[10] Antoine JC, Camdessanche JP (2004). Les neuropathies périphériques paranéoplasiques. Rev Neurol (Paris).

[11] Antoine JC, Mosnier JF, Lapras J, Convers P, Absi L, Laurent B (1996). Chronic inflammatory demyelinating polyneuropathy associated with carcinoma. J Neurol Neurosurg Psychiatry.

[12] Griggs JJ, Commichau CS, Rapoport AP, Griggs RC (1997). Chronic inflammatory demyelinating polyneuropathy in non-Hodgkin's lymphoma. Am J Hematol.

[13] Wada M, Kurita K, Tajima K, Kawanami T, Kato T (2003). A case of inflammatory demyelinating polyradiculoneuropathy associated with t-cell lymphoma. Acta Neurol Scand.

[14] Tse E, Kwong YL (2013). How I treat NK/T-cell lymphomas. Blood.

